# Exergaming (physically active video gaming) for mental health service users in a community mental health care setting: an ethnographic observational feasibility study

**DOI:** 10.1186/s12888-023-05233-6

**Published:** 2023-10-16

**Authors:** Seren Haf Roberts, Jois Bailey

**Affiliations:** 1 School of Healthcare Sciences, College of Biomedical and Life Sciences, Cardiff University, Heath Park, Cardiff, CF144XN UK; 2 Betsi Cadwaladr University Health Board, Wrexham, UK

**Keywords:** Ethnography, Exergaming, Mental illness, Physical activity, Community mental health

## Abstract

**Background:**

People with severe and enduring mental illness experience health inequalities with premature mortality; lifestyle behaviours are known to be contributing factors with low levels of physical activity reported. Facilitating physical activity to help maintain or improve health for those who are disadvantaged is essential. Exergaming (gaming involving physical movement) is increasingly used to improve physical activity across the lifespan and for those with a range clinical conditions; this might offer a way to increase physical activity for those with severe mental illness. The aim of this study was to explore engagement of mental health service users with exergaming to increase physical activity in a community mental health care setting.

**Methods:**

An ethnographic observational feasibility study was undertaken through participant observation and semi-structured interviews. A gaming console was made available for 2 days per week for 12 months in a community mental health setting. A reflexive thematic analysis was performed on the data.

**Results:**

Twenty one mental health service users engaged with the intervention, with two thirds exergaming more than once. One participant completed the semi-structured interview. Key themes identified from the observational field notes were: support (peer and staff support); opportunity and accessibility; self-monitoring; and perceived benefits. Related themes that emerged from interview data were: benefits; motivators; barriers; and delivery of the intervention. Integrating these findings, we highlight social support; fun, enjoyment and confidence building; motivation and self-monitoring; and, accessibility and delivery in community mental health care context are key domains of interest for mental health care providers.

**Conclusions:**

We provide evidence that exergaming engages people with SMI with physical activity. The value, acceptability and feasibility of open access exergaming in a community mental health service context is supported. Facilitating exergaming has the potential to increase physical activity for mental health service users leading to possible additional health benefits.

## Background

Physical and mental health benefits of undertaking regular physical activity is established for people with severe mental illness (SMI) [1–3], yet as many as 72% of the individuals with SMI do not meet recommended physical activity guidelines [4] set out for general population health [5]. Mishu et al. (2019) found that only 38% of adults with SMI reported undertaking regular physical activity [6]. Meanwhile, Martland et al (2023) reported that only 29% of their sample with SMI completed 150 minutes or more of moderate and/or vigorous activity per week, with 72.2% spending more than 6 or more hours per day sitting [1].

Sedentary lifestyle and the lack of physical activity has been identified as a contributing lifestyle or modifiable risk factor for poorer physical health experienced by people with SMI, particularly in relation to cardiometabolic and cardiovascular risk factors [1, 7–10], leading to increased risk of premature mortality [11] and poorer physical health outcomes compared to the general population [12]. Importantly, 61% of participants with SMI in one study reported that they wanted to undertake more physical activity and wanted to be supported to so [6]. Moreover, a qualitative study exploring the views of participants with SMI about an educational lifestyle intervention found that participants wanted a structured physical activity programme to run alongside the educational component of the intervention [13]. 

Nevertheless, barriers to physical activity engagement remain for this clinical group [3, 4, 14, 15]. Qualitative research has highlighted the important role of social support from staff and peers in engaging people with SMI with physical activity [16]. Firth et al., (2016a) also identified the lack of support as a significant barrier to engaging patients with mental health conditions with exercise; along with other barriers including stress, depression, low energy, lack of training partner, and lack of time [15]. To increase physical activity for people with mental illness, they suggest that professional support and supervised interventions would be beneficial; while Soundy et al. (2014) acknowledged that group context offering social support may also increase engagement with physical activity [16]. 

New generation gaming consoles are increasingly used for health benefit [17], in particular to increase physical activity [18] in children and adolescents, [19–22] as well as adults [23, 24] and older adults [25, 26]. Known as exergaming, this type of gaming has been shown to increase energy expenditure [27, 28], physical activity [29], and fitness [30]. According to Oh and Yang (2010) “Exergaming is an experiential activity where playing exergames, videogames, or computer-based is used to promote physical activity that is more than sedentary activities and also includes strength, balance, and flexibility activities … exergaming is part of playing video games for a healthy lifestyle” (pg 9) [31]. 

Clinical applications of exergaming have also been explored, with increasing use to support patients with a variety of health conditions across the lifespan such as musculoskeletal [32]; neuromuscular [33]; cancer [34]; dementia, [35]; parkinson disease [36]; neurodevelopmental and psychiatric conditions [37]; and, depression [38]. This growing body of evidence for active gaming for health, and its utility within healthcare, highlights the possible benefits; yet few studies have explored the physical and mental health benefits of exergaming with people with SMI within mental health services. Only one recent study protocol was found in this area with no findings yet published [39]. 

With these findings in mind, this study was designed to explore engagement of adults with SMI, alongside their carers or support workers, with an open access exergaming programme set in a community mental health care setting to increase their physical activity. Our objectives were to a) observe the uptake of exergaming amongst mental health services users; b) explore users’ experience and acceptability of accessing exergaming within community mental health care setting; and, c) determine the feasibility of facilitating physical activity in the form of exergaming in this context. 

## Methods

An observational prospective feasibility study was undertaken, underpinned by an ethnographic approach [40], using observation and informant interview techniques to explore engagement with exergaming in a community mental health care setting. An ethnographic approach was used to gain understanding of the cultural and behavioural dimensions around this intervention. Community mental health care is typically multidisciplinary and is effective in providing care and treatment to people with SMI outside hospital environments [41–43]. The community mental health setting for this study was a central building housing a multi-disciplinary team within a large town (local authority with population of about 136,0000), from where mental health care and treatment was delivered to those SMI living within the local community. 

### Intervention

An exergaming console was made available to service users and their carers or support workers for 12 months within a community mental health care setting with open access for two full days per week. The console required no controllers to minimise likely injury or damage, and the gaming sensor detects full body movement without the need for any equipment. A range of physically interactive games were available to suit a range of interests, abilities and fitness levels. Drinking water was always available; and clear warm up and cool down instructions were provided along with advice on overexertion and injury. Observations and, with consenting participants, semi-structured interviews were undertaken. 

### Setting

The community mental health care setting was a community rehabilitation team (CRT) serving 2 counties (both with urban and rural areas) with a combined population of 288,000. Each county also had an Adult CMHT, Child and Adolescents CMHT, Substance Misuse CMHT and Older Adult CMHT. The CRT was a multi-disciplinary integrated health and social care service providing support to people with severe and enduring mental illness with complex needs and a predominantly forensic history. The team had approximately 10 care coordinators with a case load of between 20 – 30 during the study time frame, and about 30% of these cases were residing in independent hospitals and therefore unable to engage with the study. 

To maximise the space available for the intervention, the gaming console was permanently set up at one end of a large meeting room on the ground floor of the setting with a large 50” flat screen TV. All games were available in the room and stored close to the console. Furniture were pushed against the wall to provide space during the sessions. The room was made available for console use on 2 days per week and used for usual CMHT operational needs and team meetings for the remaining 3 days. 

### Observation

Use of the console was monitored and the physical and social activity around the console were observed and recorded in field notes. Participant observation was undertaken by a Healthcare Support Worker employed within the Community Mental Health Team and was independent from the research team. The participant observer was provided training in research observation and data collection and facilitated access to the console, observed and monitored activities in and around the console, and kept descriptive field notes with some reflective accounts for a period of 12 months. The nine dimensions of observation underpinned the observation [44]. Observation was overt and made known to all console users. Consent was not required for this aspect of the study in line with ethical approval. For this reason, personal information such as diagnoses and length of time engaging with mental health services were not recorded about those using the console. 

### Qualitative interviews

All individuals who accessed the console were invited to participate in a one-to-one semi-structured interview at a convenient time within the community mental health care setting to explore their experience with the console in more depth. Written informed consent was obtained for this aspect of the study and additional demographic data were collected for interview participants in line with ethical approval. 

### Sampling and recruitment

**Fig. 1:**
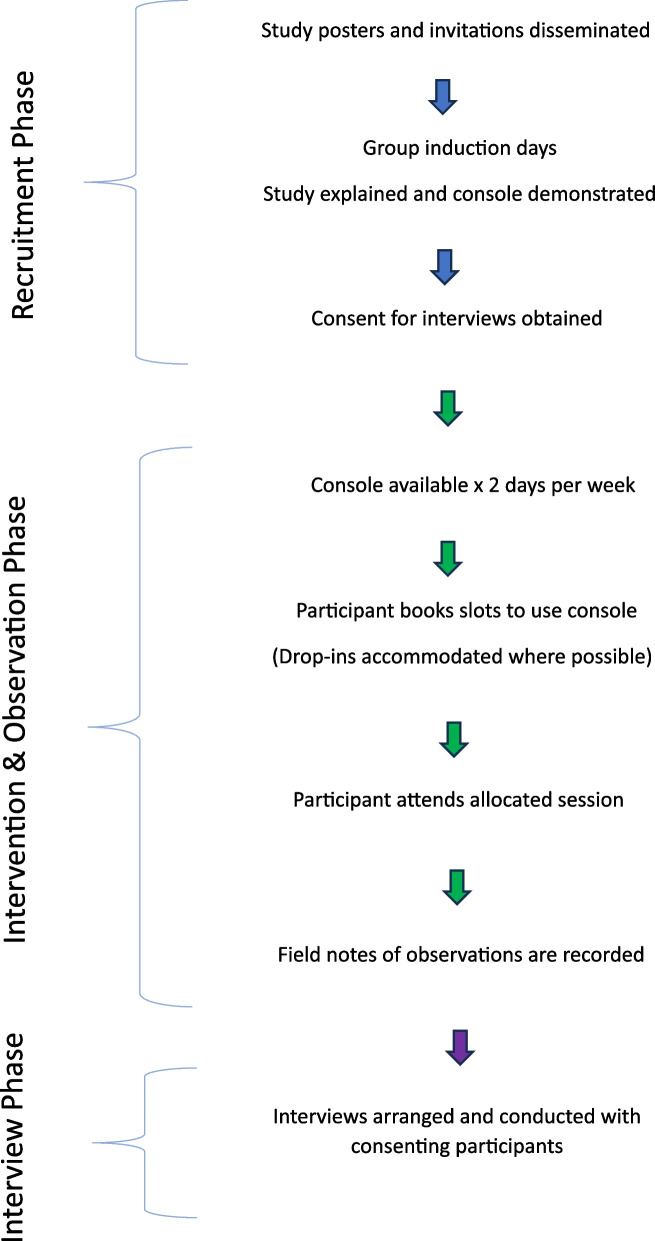
Study flowchart

The console was open to all mental health service users and their carers (including support workers) in the local area. Posters about the console availability were disseminated to all community and inpatient mental health teams in the area along with an invitation letter to inform them of the study and ask if they would bring the study to the attention of their service users, carers and support workers. The poster invited interested service users, carers or support workers to contact the research team. Distribution was staggered over time to gauge likely response and allow management of the demand if required. A study information sheet was sent and an induction meeting arranged with anyone making contact with the team. At the induction meeting a member of the research team oriented the individual to the equipment; gave a demonstration of the console; provided safety information about the equipment, safe exercising and injury prevention; and, discussed the study. Please see study flowchart (Fig. 1).

### Analysis

As a feasibility study, engagement with the intervention (exergaming) was of primary interest. Descriptive analysis of the observational field notes was undertaken in relation to attendance and use of the console. A reflexive thematic analysis [45] was undertaken of field notes to explore the culture and behaviours around the console, and of interview data to explore perceptions and experiences. Repeated reading and coding of the field notes and interview transcript were undertaken independently by the lead author and verified by the second author. Agreement and consensus of codes and themes were confirmed through discussions. Once the generated themes were agreed, they were described and summarized with quotes selected as illustrations. 

### Ethics, rigour, trustworthiness and reflexivity

Ethical approval for the study was obtained from an NHS Ethics Committee. Although assessing rigour of qualitative research continues to be debated, issue of trustworthiness, especially transparency, must be considered to demonstrate quality in the evidence generated [46, 47]. For this study, we adhered procedurally to established data collection and analysis methods in qualitative research, specifically ethnography; and, maintained accurate records of the research process, which are clear, transparent, and auditable. Semi-structured interviewing techniques, independent participant observer, and independent coding of data maintained some objectivity. Observations were overt to ensure service users felt comfortable and to avoid participant distress. Data-source triangulation [40] was also undertaken to ensure credibility and integrate findings. 

Whilst these procedures afford some objectivity, the risk of subjective bias was possible thorough the authors’ knowledge of a) the study setting (i.e. the community mental health service); b) many of the service users; and, c) the lifestyle issues of the client group previously observed in their role in health promotion activities within this service. These were considered during the design, data collection, and analysis process through reflexive thinking [48] and discussions. The subjective knowledge might allow for better understanding and interpretation of the data because of this underpinning knowledge. Whether services users’ relationships with the authors and participant observer impacted engagement and behaviour was considered; but, the authors had minimal input into the intervention delivery and observations and thus would not have been visible to service users during their engagement with the intervention, and the participant observer played no part in the analysis, thereby maintaining some independence of process. Similarly, the fact that the intervention and overt observation was facilitated and recorded (independently form the authors) by an established member of the community mental health team may have influenced service user engagement and impacted on their behaviours was also considered; however, it was determined that prior relationships are more likely to have enabled service users to feel more comfortable and thereby reflect their natural behaviours during their engagement. The challenges experienced with recruiting informant interviewees also indicates that prior therapeutic relationships did not provide undue influence on participation or engagement. 

## Results

Twenty one individuals with a SMI and known to the CMHT hosting the console engaged with the gaming console at least once with 1 participant taking part in a semi-structured interview. 

### Descriptive results from observational field notes

Fourteen (66.7%) of the 21 service users who used the console attended two or more times. Of these, 6 (43%) attended more often by themselves than with another person (µ= 11 visits; range 2 – 36 visits); 2 (14%) service users attended twice by themselves and twice with another person; and the remaining 6 (43%) attended more often with another person (µ= 6 visits; range 3 -8). Almost half of the service users (*n*= 9; 43%) attended 5 sessions or more. Two service users were highly regular console users with one attending 42 sessions and another attending 22 sessions across the time frame. For 14 of the prearranged sessions, 11 did not attend (DNAs), 1 cancelled with no reason given, 1 cancelled owing to repair contractors at home, and 1 was unwell. 

The field notes highlight that a bowling game was the most popular game (recorded use *n*= 40) with a dancing game second (recorded use *n*= 17). With more exposure, users became more confident to try other games, which would be introduced during the sessions. 

A third of those who engaged initially did not continue to engage in subsequent sessions, attending just once during the study period. As an observational study, available data are limited; despite invitations to participate in interviews to explore the barriers to attendance, none agreed. Possible reasons for this non-engagement are explored in the discussion. 

### Thematic analysis of observational field notes

Key themes generated from the qualitative thematic analysis of the field notes are shown in table 1.

**Table 1:** Themes from observational field notes analysis

**Themes**	**Subthemes**
Support	Encouragement and peer support Staff support and involvement Motivation Social networks
Opportunity and accessibility	Arranging access Pursuing other physical activity Preparedness
Self-monitoring of progress	Previous scores Others’ scores
Perceived benefits	Enjoyment Confidence Getting out of the house Weight loss

#### Support

Several service user seemed to prefer, and were more motivated, to play with others and encouraged each other when playing together. One service user “*gave encouragement to the others when they had their turn at bowling and all three gave each other a ‘high five’ when they scored a strike*.” (Field note transcript 63). However, the service users also enjoyed the social context and would happily chat instead of engaging with the game without prompting by the facilitator. “*Staff did have to encourage them to continue with the game after they had finished their drinks. All three were happy to continue talking rather than be interested in bowling”* (Field note transcript 45). One service user was asked if another could join in her session and reported that “*it was a good idea and found it better if she played the game with someone else.”* Both later reported that *“they had enjoyed the session and the company of the other*” (Field note transcript 41). 

Preference for playing with others was also highlighted with service users often asking staff to help set up the games, even after repeated use, and participate in the games. They seemed to particularly enjoy playing with staff and in doing so they were seen to *“interact well with staff and laughed when they missed”* (Field note transcript 39). Service users liked support from staff reporting that they *“enjoyed more with staff”* (Field note transcript 35) and found it *“less boring with staff”* (Field note transcript 34). This suggests that the social context is at least as rewarding as the physical activity and that the activity within a community mental health environment enabled a continued connection with staff, and perhaps services, that the service user found helpful. 

#### Opportunity and accessibility

Most sessions were arranged by the service user in advance typically arranging a regular slot; although occasionally service users would turn up opportunistically. With more frequent use, service users came more prepared for the physical activity in appropriate clothing (tracksuits and trainers) and bringing drinks along (sometimes energy drinks) with them, although water was available at all times. This suggest that for at least those who used the console regularly, they considered it a form of exercise. 

Some service users found the equipment difficult to use. *“[Service User] found using the hand sensor difficult and was not able to use the voice activation as she did not know what songs were available”* (Field note transcript 40). Some service users would engage for a single session but with inappropriate attire such as *“casual clothes with a large coat”* (Field note transcript 72) and then because they found it difficult to operate the console, did not seem to enjoy the experience. However, others, particularly with more frequent use, would only require verbal prompting to set up the game and in some cases became confident enough to set up the game entirely independently. 

#### Self-monitoring of progress

Services users enjoyed the competition when playing with others. As well as competing against others, they seemed to enjoy monitoring their own progress and would try to better their own scores. There was clear motivation to achieve personal best scores which seemed to uplift mood and bolster self-confidence. After clarifying the high score of the day, one participant stated that she had *“broken that score today I have got 230, I’ve had four strikes in a row”* (Field note transcript 39). 

#### Perceived benefits

Service users reported enjoying using the console deriving some pleasure from the activity. They especially enjoyed playing with others indicating that the social interaction, competition and company of others was beneficial. Laughter was a commonly reported observation in the field notes suggesting that the service users seemed to enjoy the camaraderie, social support and encouragement from others. One service user stated *“I really enjoyed that session”* when participating with others (Field note transcript 13). There was also a sense that the activity provided a structure and purpose by getting the service user *“out of the house and gives him something to do in the morning”* (Field note transcript 61). 

Weight loss was also a perceived benefit of the activity and provided a sense of achievement and confidence. One service user *“laughed all the way through the dance and stated that was really good, I enjoyed that”* later reporting wanting to attend *“on a regular basis as she found it fun and thought it would help with weight loss.”* (Field note transcript 40). 

The activity appeared to provide a therapeutic benefit, providing an opportunity to talk to staff, which seemed to help improve mood. One service user *“opened up to staff while bowling about how she was feeling, [Service user]’s mood began to lift during the game as staff spoke about possible solutions to her current difficulties”*; later reporting *“I feel a lot better now”* (Field note transcript 77). 

Overall, the field notes suggest that there are both physical, social and emotional benefits to engaging with the gaming console. The camaraderie, peer support and social context seem to increase the enjoyment element and was an important motivating factor. Mood and motivational improvements were also observed and it is possible the service users felt reassured with an indirect but continued contact with the community mental health team. 

### Thematic analysis of interview data

All service users engaging with the console were invited to interview with one female participant completing the interview. Four main themes were generated from thematic analysis: Benefits, motivators, barriers, and delivery (see table 2).

**Table 2:** Summary of themes from interview transcript

**Key themes**	**Descriptor**
**Benefits**	Positive experience with perceived and actual benefits reported
**Motivators**	Motivator for participant to engage and how to motivate others to participate
**Barriers**	Barrier to participants’ own engagement with the activity and barrier to others’ engagement
**Delivery**	General set up of the activity within the community mental health setting, opportunities for drop-in, staff involvement and playing against someone.

#### Benefits

The theme around benefits related to both the perceived and actual benefits as reported by the participant and was reported to be a positive experience. The participant reported that the activity provided a structure to their day or activities, helped to maintain relationships with services and staff and increased the likelihood of engaging with other physical activities such as walking more. The participant also reported physical benefits in the form of feeling fitter and losing weight as well as mental health and psychological benefits such as relieving the mind and developing self-confidence. *‘It gives you some structure to your day… keeping a relationship going with the staff here … I’d just say it motivates me to walk more… I thought it would make me fitter, which it has done… And just feeling better after doing it, I have more energy after each class I did of the [console]… it also sort of relieves my mind, it takes my mind off other things, my problems and focuses it on something else… it’s helped me with the weight loss, my confidence is improving in myself.’*

#### Motivators

This theme related to what motivated the participant to engage with the activity and factors perceived by the participant to motivate others to engage. Encouragement from the staff, involving others including staff and regular weigh-ins were reported to be strong motivators for engagement with the activity. The participant suggested that *‘word of mouth’* would likely motivate others along with a drop-in basis for the activity in which people could play against other people. *‘If you could play against someone… they normally weigh me sometimes after the [console] and that motivates me as well… Just speaking to other people who have done it and can vouch for it… It’s fun, fun, fun game, it keeps you fit, it motivates you, and I do feel better after I’ve done it, it gives me more energy for the day.’*

#### Barriers

Barriers to the participant’s own engagement with the activity and the engagement of others were identified. Limited staff support generally and in trying new games was considered a key barrier alongside staff expectations about level of engagement. As noted by the participant, a key barrier is *‘Probably having no support around. Sometimes it’s tricky working out how to put the [console] on and set the whole thing up, so you do need support with that, you do. It took me about ten minutes once. And I had an appointment afterwards so it was eating into that time… Perhaps the amount of time you have to spend on it. Yes, because for me, a good twenty minutes on it is enough sometimes’.* The participant reported that spending longer time on the activity was *‘expected of you… sometimes, it gets a bit repetitive playing the same things that you like on it… playing bowls for an hour on your own, gets a bit boring’.*

#### Delivery

This theme coalesced issues relating to the general set up of the activity within the community mental health care setting, which was viewed favourably. The activity was deemed to be easy to access with an appropriate choice of games. The participant reported maintaining her level of play with the games without changing the intensity or pushing to higher levels of the game. It was suggested that support from staff would enable service users to progress with the games. 

Whilst the participant viewed the community mental health care setting for the activity favourably *‘I prefer here. One, because I know everyone here. So I know that I can ask for help at any given time … I think it’s been set up well… and would continue to use it…’*, it was recognised that there were challenges; *‘I know it’s very awkward trying to find the time for that room to be empty, just to use the [console], so you should be given time of day, sometimes I have other appointments…’* and a drop-in opportunity was proposed. However, the participant also acknowledged that the approach used offered structure to the week *‘I turn up at a set date and time and it’s usually set up working for me, and I usually play against, on my own, you know, by myself.’*

With regard to delivery, the idea of the drop-in approach alongside having increased staff presence, engagement and support were regarded by the participant to be important potential improvements. *‘If you could play against someone… with someone though, which I prefer, to be with someone, otherwise, it can get a bit boring on your own.’*

## Discussion

### Integration of findings

Integrating the qualitative findings generated form the observations and informant interview, through data source triangulation [40], highlights four notable overarching concepts or domains as shown in table 3.

**Table 3:** Integrated findings

**Integrated domains**	**Summary**
Social support	Actual support provided by staff, peers, family and carers
Fun, enjoyment and confidence building	Intervention needs to be engaging and interesting to develop mastery, which is confidence building
Motivation and self-monitoring	Self-monitoring progress is a motivator for attendance
Accessibility and delivery in community mental health care context	Facilitating physical activity through exergaming is accessible, acceptable and valued in a community mental health setting.

With regard to social support, there was a clear and strong message from our data that indicated the important role of the social context of the intervention. The service users seemed to enjoy and more actively participate when others were also engaged; and this included staff participation. The social element seemed to draw the service users to attend regularly. This is consistent with previous research [13–16, 49] highlighting the value of social support from others in engaging people with SMI with health promoting behaviours. 

The fun and enjoyment element of the intervention was also important in enabling service users to build their confidence and assess their progress in a subtle way, which also helped motivate them to attend regularly. Enjoying exercise has been show to increase adherence [14, 15]; and Fleming et al (2017) reports that exergaming interventions need to be engaging as well as effective [50]. We have shown that for people with SMI, exergaming is a viable and engaging physically active intervention. Developing their skills and gaining mastery of both setting up the console, and the gaming skills specifically to improve their scores, helped to build confidence. This seems to act as a motivator to attend and exergame. Being able to monitor their progress through their own personal best, and also in comparison to the performance of others through competition, sustained their interest. There may also be additional health benefits of playing competitively with recent evidence suggesting that multiplayer exergaming results in higher heart rate and a tendency for increased oxygen uptake over single-player exergaming [51]. 

The availability, accessibility and community mental health care context was also important. Service users seemed to like attending the community mental health setting, perhaps because it was a familiar venue with familiar faces, a place where they may feel more comfortable. It may also provide the opportunity for them to maintain a link with mental health services, which may be a reassuring and motivating factor. There is limited research on mental health service users’ preferences in relation to undertaking physical activity within the context of mental health services. Previous research utilise existing community leisure schemes to provide physical activity programmes for people with SMI [14]. This study has demonstrated that service users will engage with physical activity in a community mental health context. 

This ethnographic feasibility study set out to explore the acceptability, value and utility of open access exergaming for mental health services users in a community mental health care setting to increase physical activity. To our knowledge, this is the first example of a study to explore exergaming as a way to increase physical activity for this population. Additionally, this study is the first example of the novel use of a community mental health care setting to engage service users with physical activity on site, thereby providing new insights into opportunities within healthcare systems to maximally engage people with SMI with physical activity. 

Our observations demonstrate that there was engagement with the activity with many service users attending regularly to use the console. One participant engaged fully with the intervention for the duration of the study. Other regular users seemed to engage well with the intervention, coming more prepared and getting increasingly confident in using the equipment over time. A third of participants did not engage beyond one session. Whilst determining the barriers to attendance for these individuals was limited to observational data, we believe the barriers related to 1) their relationship with the CMHT; 2) acuity of illness or illness symptoms; 3) not enjoying the gaming approach or games available; 4) attending in unsuitable attire making the activity more challenging; and, 5) lack of support from others (peers or support staff). 

Our qualitative findings provide further evidence that the group context and social support from staff and peers incentivise people with SMI to engage with physical activity, as demonstrated by others [14–16]. Furthermore, our findings support the position that structured and supervised physical activity [14, 15] also motivates engagement for these individuals. We believe that the social context and contact with staff is a critical engagement factor for any form of physical activity with this population. 

What this study adds to the evidence base is that 1) exergaming can engage mental health service users and is a feasible intervention to enable them to be more physically active; and 2) mental health services can facilitate this form of physical activity within a community mental health context. The need for exercise interventions to be incorporated in to routine care of people with mental disorders because of the known physical and mental health benefits has been previously proposed [3], and we provide the evidence for one way in which this could be achieved. A community mental health setting is a place where people with SMI are familiar with and thus feel comfortable and safe, increasing their likely attendance and engagement. This setting also provides a multidisciplinary context to further enhance engagement and sustainability of a physical activity intervention, which is needed to overcome patients barriers and enhance adherence and benefits [3]. The feasibility and viability of this setting utilising exergaming to increase physical activity of their client group has been demonstrated by our study. 

Exergaming may provide value for money; the cost effectiveness should be considered in future research. The initial outlay for the equipment was around £600 (including all games, monitor and console). The more challenging aspect of delivering this intervention was the need for space and staff time to facilitate it. The team made available their large multi-function meeting room (on days when the room was not being used for scheduled meetings) and a Health Care Support Worker (HCSW). The HCSW facilitated the bookings, prepared the room, ensured water was available, supported the setup of the equipment for participants, and participated when possible. The console was standalone and did not require wifi; therefore online gaming was not available. An itinerary of equipment was maintained with equipment secure in its location. The room was also in a secured area (behind a keypad door) and could only be accessed with staff assistance. No injuries, breakages or thefts occurred across the 12 months. Console use could be offered with minimal staff input with only practical and maintenance input. As users became more confident, the HCSW played less of a role in the exergaming, allowing the participants to set up and undertake the activity independently; however, our findings indicate that users preferred and requested staff participation, and that staff participation is a motivating factor. Wider implementation within CMHTs would need to take into account the need for dedicated space and staff to support the practical and engagement aspects of the intervention. 

Improving the physical health outcomes for people with SMI has been a national and international priority for some time [52–55]; however, there are limited documents that offer care providers the tools to realise these ambitions. Identifying ways to engage people with SMI to undertake more physical activity is one strategy to help achieve this, given the tendency for sedentary behaviour and consequent health disadvantage. Our study provides evidence of the feasibility of, and a practical example of, how mental health services can facilitate physical activity for mental health service users. We demonstrate that increasing physical activity can feasibly fall under the auspices of mental health services and that exergaming is a viable physical activity that could potentially extend to other mental health settings such as forensic mental health care, where evidence for physical activity for those with SMI is scarce [56]. 

### Limitations

This study was an observational study to explore feasibility of open access provision of exergaming in the community setting. Our study demonstrated the feasibility and value of using exergaming to increase physical activity of mental health service users, but did not set out to quantitatively examine or measure whether 1) there was an observed increase in physical activity outside the intervention; 2) any physical or mental health benefits were achieved; and 3) exergaming was superior to other forms of physical activity interventions for people with SMI. Neither the effectiveness nor efficacy of the intervention for increasing energy expenditure, weight loss, fitness, or other health and physical activity indicators, such as heart rate and oxygen uptake, were evaluated. The observational element successfully demonstrated the potential value of the intervention in this setting, but recruitment and retention of participants was a key issue in limiting the representativeness of the sample. Eight console users initially agreed to participate in semi-structured interviews with six providing signed consent forms, but following repeated non-attendance at interview appointments, we were only able to arrange this with 1 individual. Strategies to enhance recruitment of people with SMI is needed for future related research. Availability of the intervention being on one site for 2 days per week, owing to space requirements, may also have impacted on the comprehensiveness of the data gathered, limiting representativeness and transferability of the findings. Future work might take into account a full week of console availability to maximise the opportunity for engagement. This requires space, commitment and coordination, but is not insurmountable for multidisciplinary community mental health teams to consider. Additionally, application in multiple community mental health care settings would also be beneficial for future work increasing transferability. 

### Future directions for research

Future research should focus on demonstrating effectiveness and cost-effectiveness of exergaming on all the key physical and mental health indicators for this population through a randomised controlled trial, as well as investigating the impact on physical activity outside the intervention. Multiple community or mental health sites are needed, and dedicated space is required for the activity to be accessible across the full working week to maximise its potential use for this population. It is also important to further explore through both quantitative and qualitative research, the role of mental health services and personnel in delivering exergaming to maximise engagement. Research on how the multidisciplinary composition of community mental health settings can maximise opportunities to engage people with SMI in physical activity is also needed. 

### Future directions for clinical practice

Notwithstanding the need for dedicated space and equipment (including maintenance), community mental health settings can provide opportunities to engage people with SMI with physical activity in a way that 1) provides regular contact with staff, which seems to reassure service users; 2) provides a social context, which seems to motivate engagement; and 3) forms part of the routine care for individuals with SMI. Mental health services should provide a conducive environment for their service users to engage with health promoting activities, and exergaming is one example of how this might be achieved. 

## Conclusions

This study provides evidence that people with SMI will engage with exergaming, a form of physical activity. The study has also demonstrated the potential value, acceptability and feasibility of open access exergaming in a community mental health service context. Multidisciplinary community mental health services, should consider ways in which they can incorporate exergaming into their facility because it has the potential to increase physical activity for mental health service users, leading to possible additional health benefits, and this setting incentives their engagement. 

## Data Availability

The datasets used and/or analysed during the current study are available from the corresponding author on reasonable request.
